# Evaluation of Cartilage Integrity Following Administration of Oral and Intraarticular Nifedipine in a Murine Model of Osteoarthritis

**DOI:** 10.3390/biomedicines11092443

**Published:** 2023-09-01

**Authors:** Viktorija Aleksiuk, Justinas Baleisis, Gailute Kirdaite, Ilona Uzieliene, Jaroslav Denkovskij, Paulius Bernotas, Tatjana Ivaskiene, Ali Mobasheri, Eiva Bernotiene

**Affiliations:** 1 Department of Regenerative Medicine, State Research Institute Centre for Innovative Medicine, 08406 Vilnius, Lithuania; ilona.uzieliene@imcentras.lt (I.U.); jaroslav.denkovskij@imcentras.lt (J.D.); paulius.bernotas@mf.stud.vu.lt (P.B.); tatjana.ivaskiene@imcentras.lt (T.I.); ali.mobasheri@oulu.fi (A.M.); eiva.bernotiene@imcentras.lt (E.B.); 2 Department of Biomodels, State Research Institute Centre for Innovative Medicine, 08406 Vilnius, Lithuania; justinas.baleisis@imcentras.lt; 3 Department of Experimental, Preventive and Clinical Medicine, State Research Institute Centre for Innovative Medicine, 08406 Vilnius, Lithuania; gailute.kirdaite@imcentras.lt; 4 Research Unit of Health Sciences and Technology, Faculty of Medicine, University of Oulu, 90014 Oulu, Finland; 5 World Health Organization Collaborating Center for Public Health Aspects of Musculoskeletal Health and Aging, Université de Liège, B-4000 Liège, Belgium; 6 Department of Joint Surgery, First Affiliated Hospital of Sun Yat-sen University, Guangzhou 510080, China

**Keywords:** osteoarthritis, mouse, cartilage damage, meniscus, calcium, L-type channel blockers, nifedipine, biomarkers

## Abstract

Osteoarthritis (OA) ranks as the prevailing type of arthritis on a global scale, for which no effective treatments are currently available. Arterial hypertension is a common comorbidity in OA patients, and antihypertensive drugs, such as nifedipine (NIF), may affect the course of OA progression. The aim of this preclinical study was to determine the effect of nifedipine on healthy and OA cartilage, depending on its route of administration. In this study, we used the destabilization of medial meniscus to develop a mouse model of OA. Nifedipine was applied per os or intraarticularly (i.a.) for 8 weeks to both mice with OA and healthy animals. Serum biomarker concentrations were evaluated using the Luminex platform and alterations in the knee cartilage were graded according to OARSI histological scores and investigated immunohistochemically. Nifedipine treatment per os and i.a. exerted protective effects, as assessed by the OARSI histological scores. However, long-term nifedipine i.a. injections induced the deterioration of healthy cartilage. Lubricin, cartilage intermediate layer matrix protein (CILP), collagen type VI (COLVI), CILP, and Ki67 were upregulated by the nifedipine treatment. Serum biomarkers MMP-3, thrombospondin-4, and leptin were upregulated in the healthy groups treated with nifedipine, while only the levels of MMP-3 were significantly higher in the OA group treated with nifedipine per os compared to the untreated group. In conclusion, this study highlights the differential effects of nifedipine on cartilage integrity, depending on the route of administration and cartilage condition.

## 1. Introduction

Aging is associated with the development of various chronic diseases, in which both hypertension and OA are dominant. According to the latest epidemiological data, more than 500 million people have OA worldwide [1], with disease prevalence increasing with age and obesity. OA affects all joints in the body and their parts, including the cartilage, synovial membrane, infrapatellar fat pad, meniscus, and subchondral bone. At present, there is no effective pharmacological treatment for OA and there are no prognostic biomarkers for the evaluation of disease progression and follow-up of treatment effects, which highly complicates the development and clinical evaluation of novel therapies [2].

Arterial hypertension is another huge age-related healthcare problem worldwide, and the current therapeutic agents for the treatment of hypertension include drugs that target L-type voltage gated calcium channels (VGCC), particularly 1,4-dihydropyridines [3]. The classical drug in this group is nifedipine, working through VGCC subunit CaV1.2 and thereby reducing blood pressure [4].

Recent work has demonstrated that chondrocytes express multiple VGCC isoforms, which are important for mechanotransduction. Moreover, the biomechanical behavior of OA chondrocytes is different compared to healthy chondrocytes [5]. During the course of OA, the entire joint structure undergoes degradation, including the articular cartilage, subchondral bone, synovial tissue, and meniscus [6]. Normal mechanical loading plays a vital role in sustaining and supporting the physiological function of chondrocytes [7,8]. As hypertension and OA are prevalent in the older population, especially those who are overweight and with metabolic disorders, the use of antihypertensive drugs may have additional effects on the articular cartilage and other osteoarticular tissues. Studies suggest that the relationship between OA and arterial hypertension is more complex than just age and obesity, also incorporating sex, environment, and concentration of hormones in the serum [9]. Elevated blood pressure has the potential to raise intraosseous pressure, leading to a subsequent induction of hypoxia. This, in turn, initiates the process of remodeling in the subchondral bone and osteochondral junction [10]. Higher blood pressure was associated with a worse radiographic grade of knee OA [11]. Moreover, alterations in the ion channels that enable Ca^2+^ transport across the plasma membrane seem to be critical for cartilage degeneration in OA [12,13,14,15]. As hypertension is clinically treated with VGCC inhibitors, while Ca^2+^ signaling through VGCC is crucial for mechanotransduction in the cartilage, it is important to understand the mechanism behind hypertensive drug action on articular cartilage function and metabolism, and to determine whether anti-hypertensive drugs play a role in the onset and progression of OA. However, the role of VGCC inhibitors in the pathogenesis of OA has not yet been clarified. Although they can inhibit chondrogenesis in vitro, they can also exert beneficial effects by reducing the catabolic degradation of the extracellular matrix (ECM) and promote cartilage hypertrophy [16,17,18]. These studies also highlight the importance of chondrocyte homeostasis; it may be possible that low doses of VGCC inhibitors stimulate cell metabolism, but inhibit it at high doses.

ECM degradation, proteoglycan loss, and the disorganization of collagen fibers occur in OA, and the superficial layer of the cartilage is destroyed by increased friction between the articular surfaces [19]. One in vivo study, based on a questionnaire, shows a positive clinical effect of OA in people with arterial hypertension treated with VGCC inhibitors [20]. Oxidative stress, apoptosis, and inflammation are all involved in the pathogenesis of OA, and nifedipine may regulate all of those factors and lead to the remission of OA [21]. Some in vitro studies have shown a positive effect of VGCC inhibitors on chondrocytes OA cartilage [18,22,23,24]. An in vivo study with murine adjuvant-induced arthritis has shown that L-type calcium channel antagonists, such as nifedipine, nimodipine, and nisoldipine, slow down the progression of rheumatoid arthritis through the inhibition of the synovial fibroblast type A cells and type B cells that participate in the inflammatory process [25]. Therefore, we sought to investigate the effects of long-term treatment of hypertension with calcium blockers on the course of OA in vivo [26,27].

The meniscus plays a critical role in distributing loads, providing lubrication, and ensuring stability within the knee joint, while the loss of its integrity has a critical role in OA initiation and progression [28]. Meniscal tear leads to reduced shock absorbance, which results in mechanical overload in cartilage [28,29,30]. This is associated with increased calcium flow through VGCC and altered intracellular signaling, which in turn cause the destruction of ECM [24,31]. The focus of this study was to analyze the effects of nifedipine in a murine OA model, induced by the destabilization of medial meniscus, leading to the development of the cartilage mechanical overload- and stress-induced degenerative type of arthritis [32]. Alterations in the murine knee joints were evaluated using OARSI scoring [33] and immunohistochemical (IHC) markers, which correspond to different cartilage composites, including cartilage intermediate layer matrix protein (CILP) [34], chondrocytes regulator of pericellular matrix–collagen VI (COLVI) [35,36], joint homeostatic and cartilage lubricating molecule lubricin [37,38,39,40], and nuclear marker of cell proliferation Ki67 [41].

Biochemical markers to evaluate OA clinical progression in animals and humans are currently lacking [13]. To further investigate the potential systemic responses to the VGCC modulator nifedipine on chondrocytes and articular cartilage in health and disease, we measured the serum responses of leptin, TSP-4, MMP-2, and MMP-3. Leptin is involved in the regulation of inflammation and regeneration; however, OA clinical studies have so far yielded inconsistent results, suggesting a rather complex role for leptin in inflammatory conditions in humans [42]. Leptin is an adipokine, which is basically synthesized by white adipose tissue. It plays a role in the development of OA [42]. This disease has a strong association with obesity [43]. Leptin serum levels correlated with body mass index and with pain caused by OA in hands, but did not correlate with radiologic changes in the joints [44]. Higher levels of leptin were associated with the female gender, a higher body mass index, and OA progression [45,46,47]. The upregulation of the extracellular glycoprotein thrombospondin-4 (TSP-4) is observed in damaged tissues. It participates in matrix remodeling, experiencing an upregulation in cases of OA when compared to individuals with healthy joints [48], and exhibits a direct correlation with the severity and advancement of OA [46,47]. The elevation of serum TSP-4 in OA may reflect the increased production and release of TSP-4 from chondrocytes and other joint tissues. Matrix metalloproteinases (MMP) are described both in early OA human samples and in early structural OA changes animal models, and especially MMP-3 [15]. Matrix metalloproteinase-3 (MMP-3 or stromelysin-1) is a zinc-dependent enzyme. It promotes cartilage matrix degradation, vascular invasion, and osteoclast differentiation and inhibits mesenchymal stem cell differentiation [27]. In healthy individuals, the MMP-3 serum level increases after running or jumping [49]. Matrix metalloproteinase-2 (MMP-2) is a zinc-dependent enzyme, which is in the cell membrane and can be activated extra- or intra-cellularly. In humans with OA, the expression of MMP-2 in the osteochondral unit was increased in advanced stages of OA [26]. We have hypothesized that this marker will also be elevated in the serum [48,49]. Based on the available data, we hypothesize that VGCC inhibitors used to treat hypertension may modulate the Ca^2+^ responses of dynamically overloaded chondrocytes, and this affects their function, i.e., the production of ECM.

The aim of this study was to determine the effect of nifedipine on the cartilage of healthy animals and during the development of an OA model in mice following the induction of medial meniscus destabilization.

## 2. Materials and Methods

### 2.1. Animals and Model Induction

Forty BALB/c female mice were bred and housed in our local breeding facility at the State Research Institute Centre for Innovative Medicine (Lithuania). Throughout the study, the animals were cared for in accordance with the Directive 2010/63/EU of the European Parliament and the European Council of 22 September 2010 on the protection of animals used for scientific purposes. All procedures were carried out in accordance with the institutional guidelines of the European Union and were approved by the Lithuanian Ethics Committee on the Use of Laboratory Animals under the State Veterinary Service No. G2–193, 2021.10.20. Animals aged 6 weeks and weighing 20 ± 3 g were used in the experiments. Animals were maintained in an environment of controlled temperature (23 ± 1 °C). Food and water were provided ad libitum. Licensed operators carried out all of the procedures on the animals.

OA mouse model induction. Under general anesthesia, the right legs of the mice were shaved and prepared for aseptic surgery. For medial meniscus destabilization (MMD), anterior meniscotibial and medial collateral ligaments were transected [32].

Following surgery, a period of 5 weeks was allowed for OA development before administering any treatment. The treatment duration of 8 weeks was chosen to allow sufficient time for observable chronic effects and potential physiological adaptations in the mice. Kamekura et al. developed a medial meniscus destabilization (MMD) model, and this model follow-up showed that the cartilage damage score increased gradually until 12 weeks, with statistically significant differences between the moderate and severe damage scores in weeks 2–4−8 [32]. The dose of 20-mg/kg nifedipine was chosen for the study based on a previous investigation where this dosage notably reduced blood pressure, ensuring both its efficacy and comparability to the past results [50,51]. To ensure the manifestation of responses to the last dose of nifedipine treatment and that any inflammation resulting from the injury of intraarticular injection itself had subsided, we waited 1 additional week before termination of the experiment.

### 2.2. Animal Treatment Groups

**Figure 1:**
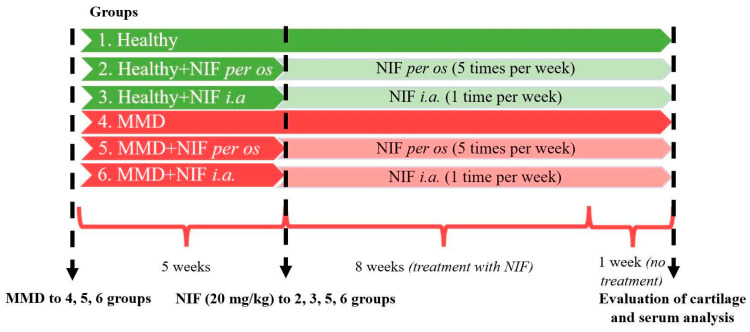
Study design: OA mice model induction and nifedipine treatment pathways. MMD: medial meniscus destabilization; NIF; nifedipine.

Treatment reagents: nifedipine powder—1 g. (>98%) (CAS-No.: 21829-25-4. Sigma-Aldrich Chemie GmbH, Germany); Dimethyl sulfoxide (DMSO)—10 mL (CAS-No.: 67-68-5. Sigma-Aldrich Chemie GmbH, Germany). The nifedipine powder was dissolved in DMSO at 50 mg/mL and the stock was diluted in phosphate buffered saline (PBS) for the treatment of 20 mg/kg, as previously described in other studies, where murine models were used [52,53]. The single dose to mice per os (orally) or i.a. (intra-articular) was 0.4 mg nifedipine in 5 µL PBS. The study design is depicted in Figure 1.

Overall, 40 mice were divided into 6 experimental groups of 6–7 animals per group:

1. Healthy (healthy animals without treatment);
2. Nifedipine per os (20 mg/kg, 5 times per week, excluding weekends);
3. Nifedipine i.a. (20 mg/kg, 1 time per week);
4. NMD (OA model group without treatment);
5. MMD+nifedipine per os (20 mg/kg, 5 times per week, excluding weekends);
6. MMD+nifedipine intraarticularly (i.a.) (20 mg/kg (1 time per week).

The first 3 groups did not undergo any procedures that might cause OA lesions; the first one served as a healthy control, the second was treated with nifedipine per os (20 mg/kg, 5 times per week, excluding weekends) (nifedipine per os), and the third by i.a. injections (20 mg/kg, 1 time per week) (nifedipine i.a.).

The fourth to sixth groups underwent surgery for MMD induction, as described above and elsewhere [15,32]. The mice in the fifth group were treated with nifedipine per os (20 mg/kg, 5 times per week, excluding weekends) (MMD+nifedipine per os), and the sixth group had i.a. injections (20 mg/kg, 1 time per week)–MMD+nifedipine i.a. Treatment duration was 8 weeks. For 1 (last) week, the mice were left without treatment.

Oral dose of nifedipine for humans is 30–120 mg/day. In the present study, the dose of 20 mg/kg nifedipine was chosen for the mice based on previous investigations where this dosage notably reduced blood pressure in murine studies, ensuring both its efficacy and comparability to the past results and clinical data [50,51]. Mice were kept after the last intraarticular injection for 1 additional week to allow for the development of responses to the last dose of nifedipine treatment, and to allow microtraumas from intraarticular injections to heal.

### 2.3. Histological and Immunohistochemical Assessment

At the end of the experiment, after 9 weeks, the animals were humanely sacrificed through cervical dislocation. The right knee was used for histological and immunohistochemical evaluation.

Histological evaluation of medial tibial plateau (MTP) of cartilage morphological changes was conducted using the OARSI (OsteoArthritis Research Society International) grading system. There are 6 grades in the OARSI semi-quantitative scoring system of mice OA, corresponding to the degree of cartilage loss: grade 1—cartilage has no loss, but staining of safranin-O is diminished; in grade 2, some superficial vertical clefts of the cartilage occur with some loss of surface lamina; in grade 3, cartilage is destroyed less than ¼ of its thickness; in grade 4, destruction of cartilage reaches ½ of its thickness, in grade 5, cartilage is destroyed by ¾ of its thickness; in grade 6, subchondral bone is denuded, so more than ¾ of the cartilage is destroyed [33]. Determination of the severity of OA in mice is effective from the single mid-coronal section, stained with safranin-O [54].

For immunohistochemistry, anti-lubricin (ab28484, Abcam, Waltham, MA, USA), anti-cartilage intermediate layer protein (CILP) (abx103807, Abbexa, Sugar Land, TX, USA), anti-COLVI (SAB4500387, Sigma-Aldrich Chemie GmbH, Germany), and anti-Ki67 (ABCAM ab9260) primary antibodies were used. Immunohistochemical staining was performed manually using the HRP/ABC detection IHC kit (ab 64264, Abcam, Waltham, MA, USA). All primary antibodies were diluted in PBS 1:200; for antigen retrieval, pepsin was used (4987481612402, Fujifilm, Tokyo, Japan). The histological preparations were photographed using an Olympus BX43 microscope (Olympus Corporation, Inc., Tokyo, Japan).

Evaluation of IHC biomarkers. Ki67 positive nuclei of chondrocytes were counted in 5 high power fields (HPF; high power field—40× magnification) and an average count per 1 HPF in each group was provided. For COLVI, we used a semi-quantitative 4-grade scoring system, depending on the intensity of the staining of the pericellular matrix of chondrocytes, where 0 stands for no labeling, 1 stands for light, 2 stands for moderate, and 3 stands for intensive labeling, using chromogen. CILP showed labeling of ECM in the intermediate layer of the cartilage. Lubricin showed surface staining and cytoplasmic staining of some chondrocytes.

### 2.4. Magnetic Luminex Assay

Serum samples from all 40 mice blood samples were collected using a standard isolation procedure, and levels of leptin, MMP-2, MMP-3, and TSP-4 were investigated using Mouse Luminex^®^ Discovery Assay 4 Plex (Thermo Fisher Scientific, Waltham, MA, USA), according to the manufacturer’s instructions. Serum samples were diluted ½ with PBS.

### 2.5. Statistical Analysis

All statistical analyses were performed using GraphPad PRISM version 9 (GraphPad, San Diego, CA, USA) and R Statistical Software (version 4.0.5; R Foundation for Statistical Computing, Vienna, Austria). Comparisons of OA grades, Ki67 positive nuclei count averages, and Luminex biomarker assay results were performed using the Wilcoxon rank sum test. Data are considered significant at a *p*-value of ≤0.05, calculated using the R and GraphPad PRISM version 9 software.

## 3. Results

### 3.1. Effect of Nifedipine Treatment on Cartilage Tissues of Healthy and Osteoarthritis Model Mice

At the beginning of the experiment, the BALB/c female mice were 6 weeks old and weighed 20 ± 3 g. Based on the weight of the mice, the dose of nifedipine was calculated to be 20 mg/kg, and was monitored throughout the treatment period. In all of the groups, the mice body weight similarly increased during the experiment.

**Figure 2:**
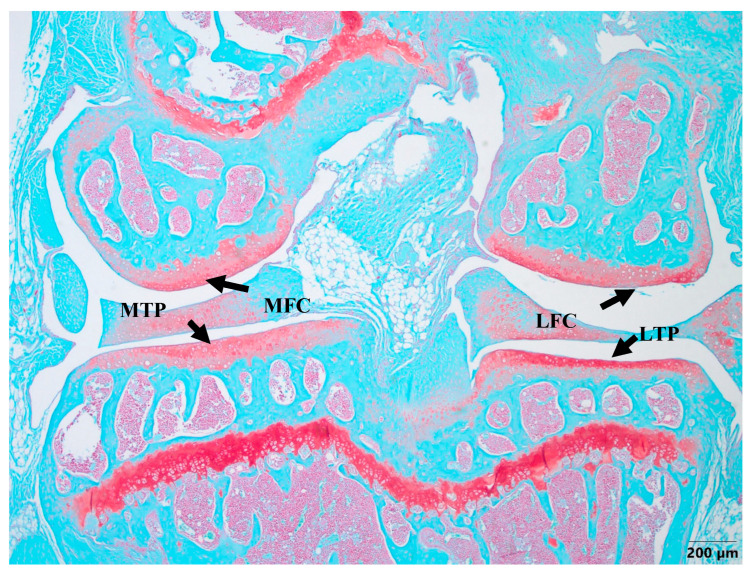
Safranin-O staining of healthy cartilage. MFC—medial femoral condyle, MTP—medial tibial plateau, LFC—lateral femoral condyle, LTP—lateral tibial plateau. The evaluation of all cartilage samples was performed in the MTP area. Magnification 4×, scale bar 200 µm.

Cartilage damage occurred in the right knee in all of the experimental groups, except the healthy group, which did not undergo any surgery and treatment (Figure 2). The healthy knee joints demonstrated the proper distribution of cells in the ECM, which was intact with a smooth superficial layer. No visual damage was observed in the cartilage of the medial femoral condyle (MFC), medial tibial plateau (MTP), lateral femoral condyle (LFC), and lateral tibial plateau (LTP) in the healthy group knees, with all areas being fully developed (Figure 2).

Histological OARSI cartilage damage was detected due to MMD in the surgically induced model, but also in the nifedipine i.a.-treated healthy control mice. Thus, in our experiment, i.a. nifedipine caused cartilage destruction signs in healthy mice.

The grade of cartilage degradation in the other groups was evaluated according to the MTP area (Figure 2) and compared to the healthy cartilage samples. The most advanced OA developed in the MMD mice without the treatment with nifedipine, while the most damaged area in the knee joint occurred in the MTP as a result of the MMD procedure being performed.

**Figure 3:**
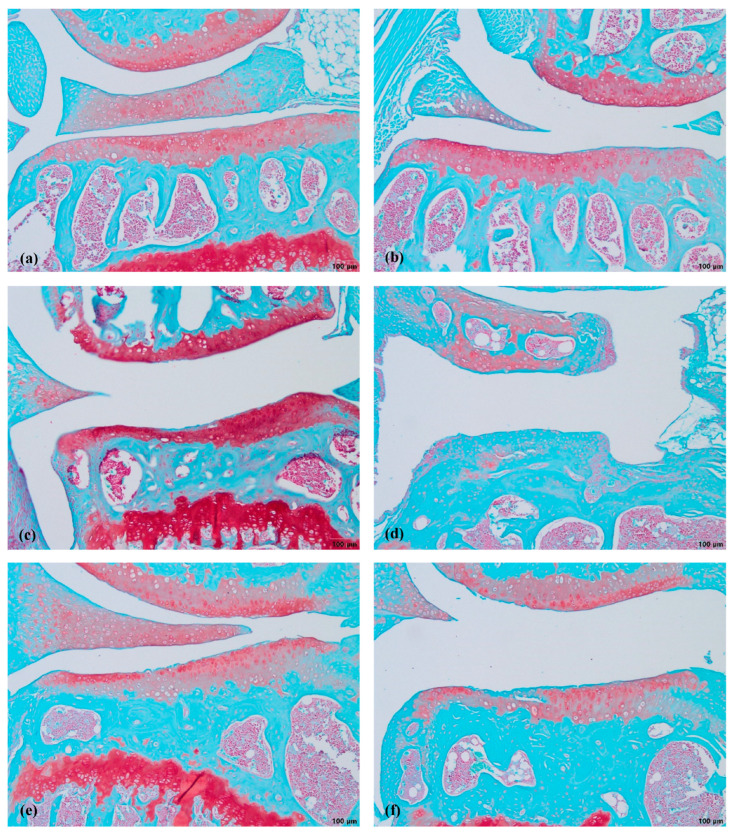
Safranin-O staining of mice cartilage. (**a**)—healthy, (**b**)—NIF per os, (**c**)—NIF i.a., (**d**)—MMD, (**e**)—MMD+NIF per os, (**f**)—MMD+NIF i.a. Arrows indicate MTP of all six groups. Magnification 4×, scale bar 200 µm.

**Figure 4:**
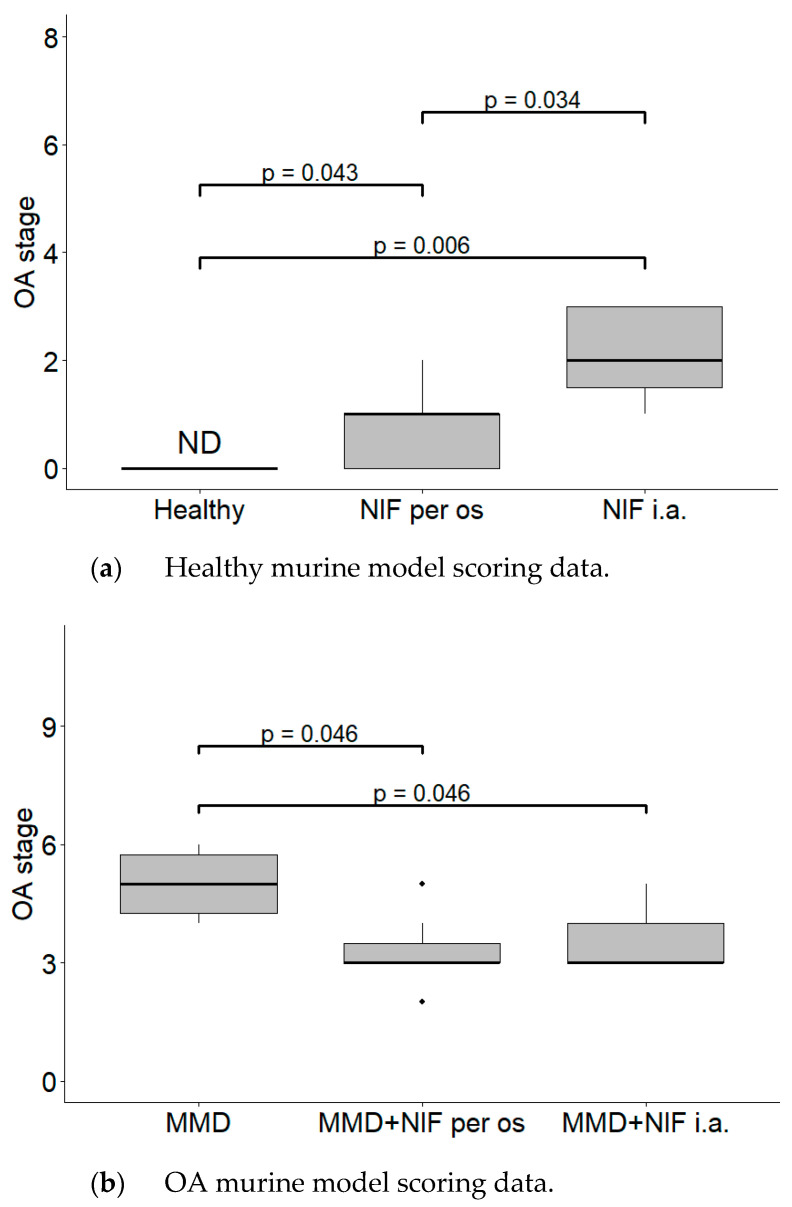
Histological changes in the mice knee cartilage tissue after the treatment with NIF. OARSI semi-quantitative OA staging system was used. (**a**) Healthy murine model scoring data. Healthy group received no treatment and had no signs of OA as evaluated using the OA staging system (ND, not detectable). NIF groups were treated with NIF for 8 weeks either per os (20 mg/kg, 5 times per week, excluding weekends), or intraarticular injection (20 mg/kg, 1 time per week). (**b**) OA murine model scoring data. Medial meniscus destabilization (MMD) was induced by the cutting of anterior meniscotibial and medial collateral ligaments. MMD + NIF groups were treated with NIF for 8 weeks either per os (20 mg/kg, 5 times per week, excluding weekends), or intraarticular injection (20 mg/kg, 1 time per week). Data is visualized using a boxplot, statistically significant differences are noted above with corresponding *p*-values, and plot points separated from the bars represent individual data. Wilcoxon rank sum test was used, data is considered significant at a *p* value < 0.05.

The MMD resulted in severe cartilage damage (up to 6 points score). The cartilage in the MMD model group had vertical clefts, and in some of the mice, reached the subchondral bone, occupying more than ¾ of the cartilage thickness. In the nifedipine per os-treated group, the cartilage surface is wavy and some superficial fibrillations are observed. However, few mice have absolutely healthy joints. The cartilage surface in the nifedipine i.a.-treated mice joints was damaged, with vertical clefts occupying ½ of the cartilage surface. (Figure 3). After the nifedipine i.a. injection, the cartilage showed more inflammatory signs in comparison to nifedipine per os (*p*< 0.05) (Figure 4a). In the mice in both of the MMD treatment groups, the OARSI scores significantly diminished and ranged between 3 points and 4 points in comparison with the MMD model group (*p* < 0.05) (Figure 4b).

The most obvious damage of the cartilage occurred in the MMD group (Figure 3f. and in Figure 4b).

### 3.2. Nifedipine Effects on Cartilage Tissue Biomarkers

The immunohistochemical detection of Ki67, COLVI, CILP, and lubricin was performed on the mice joints and the distribution patterns of these antibodies in the region of MTP were evaluated.

**Figure 5:**
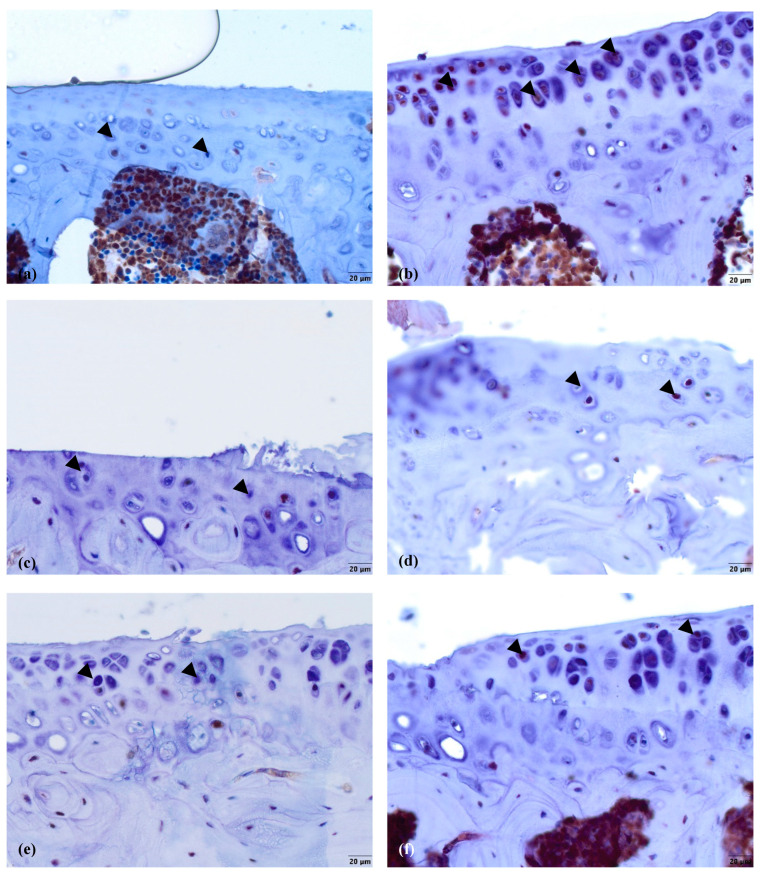
Ki67 immunostaining in murine cartilage. (**a**)—healthy, (**b**)—NIF per os, (**c**)—NIF i.a., (**d**)—MMD, (**e**)—MMD + NIF per os, (**f**)—MMD + NIF i.a. Arrowheads indicate nuclei of chondrocytes. Magnification 40×, scale bar 20 µm.

There were no signs of Ki67 in the healthy cartilage (Figure 5a), while in the other groups, some staining occurred in a fraction of the chondrocytes in the MTP (Figure 5b–f). In the nifedipine per os group (Figure 5b), even though there were no direct manipulations performed on the joint, the chondrocytes expressed Ki67. In the nifedipine i.a. (Figure 5c) group, the cartilage was damaged, but a few chondrocytes did still express Ki67 as they try to rebuild destroyed cartilage. In the MMD group (Figure 5d) without any treatment, the cartilage was destroyed, but some chondrocytes did express Ki67, as in the nifedipine i.a. group (Figure 5c). In the MMD+nifedipine per os (Figure 5e) and MMD+nifedipine i.a. (Figure 5f) groups, the expression of Ki67 in the chondrocytes is more visible, and the count of chondrocytes is higher than in the MMD group (Figure 5d).

**Figure 6:**
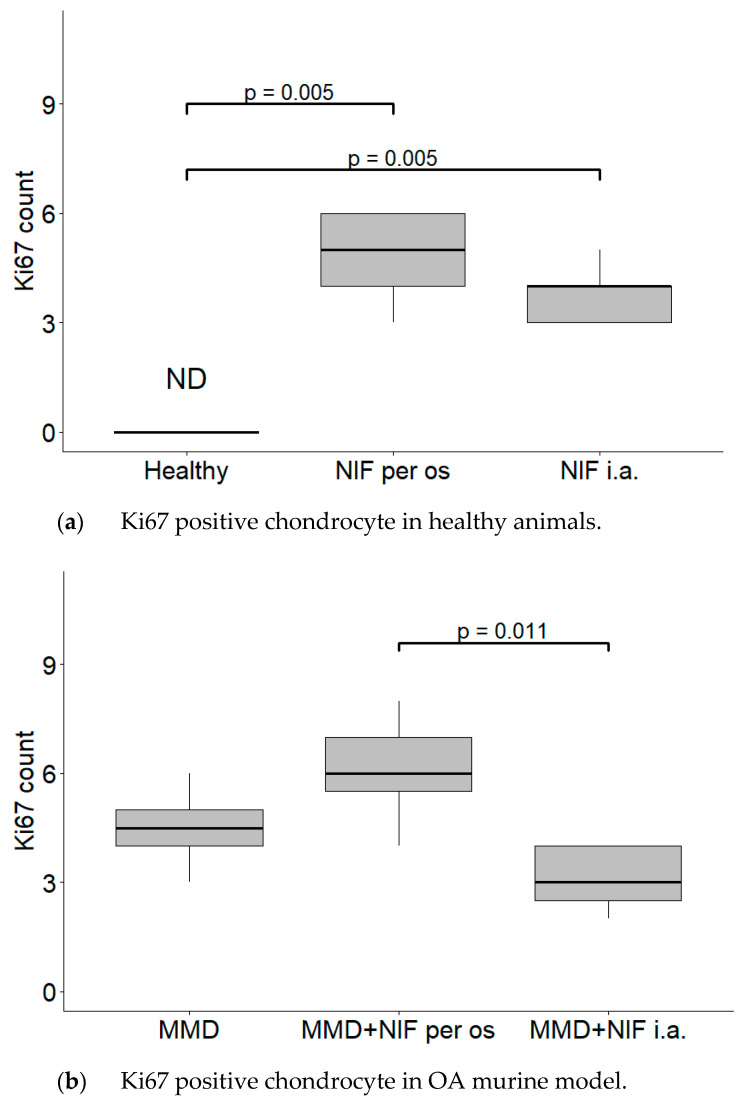
Counts of cartilage Ki67 positive chondrocyte nuclei per 1 high power field (HPF). Positive chondrocytes were counted per 5 high power fields (HPF—40× magnification), the average count of positive cell nuclei/HPF is presented in every single group. (**a**) Ki67 positive chondrocyte in healthy animals. Healthy group received no treatment and had no signs of OA as evaluated using the OA staging system (ND, not detectable). NIF groups were treated with the drug for 8 weeks either per os (20 mg/kg, 5 times per week, excluding weekends), or intraarticular injection (20 mg/kg, 1 time per week). (**b**) Ki67 positive chondrocyte in OA murine model. Medial meniscus destabilization (MMD) was induced by the cutting of anterior meniscotibial and medial collateral ligaments. MMD + NIF groups were treated with NIF for 8 weeks either per os (20 mg/kg, 5 times per week, excluding weekends), or intraarticular injection (20 mg/kg, 1 time per week). Data is visualized using a boxplot, statistically significant differences are noted above with corresponding *p*-values. Wilcoxon rank sum test was used, data is considered significant at a *p* value < 0.05.

The average count of positive cell nuclei/HPF in all six groups is shown in Figure 6. Positively stained chondrocytes were counted per 1 high power field (HPF) at 40× magnification. No Ki67 biomarker staining was detected in the healthy control group. In both groups treated with nifedipine, increased Ki-67 staining was observed (*p* < 0.05) in comparison with the healthy animals (Figure 6a). The difference between the MMD+nifedipine per os and MMD+nifedipine i.a. groups was also detected (*p* < 0.05) (Figure 6b).

**Figure 7:**
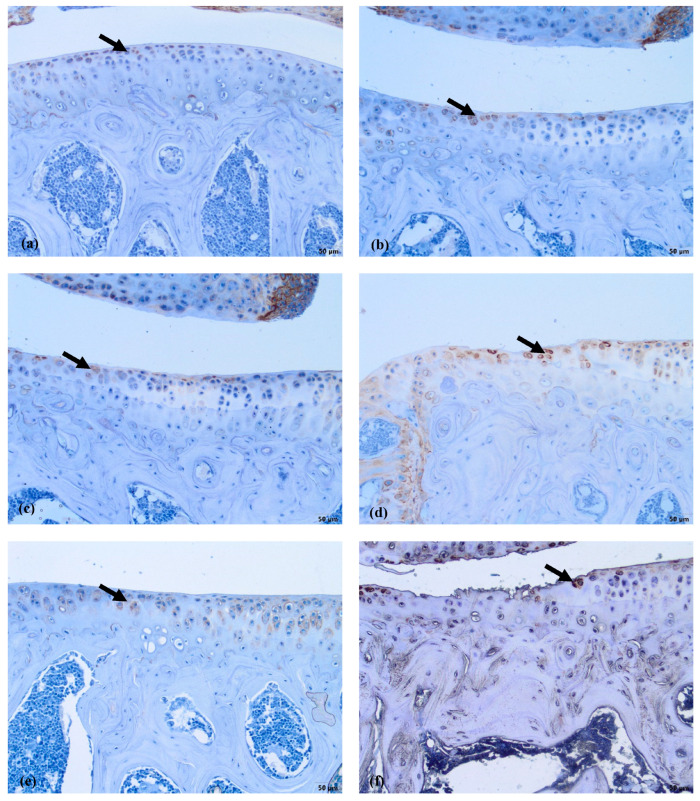
COLVI immunostaining in murine cartilage. (**a**)—healthy, (**b**)—NIF per os, (**c**)—NIF i.a., (**d**)—MMD, (**e**)—MMD + NIF per os, (**f**)—MMD+NIF i.a. Arrows indicate pericellular ECM. Magnification 10×, scale bar 100 µm.

The COLVI is located pericellularly. The expression of COLVI in OA-damaged cartilage around chondrocytes becomes more intensive (Figure 7d–f). Antibodies against COLVI intensively labeled the pericellular matrix of chondrocytes in the MMD group (Figure 7d)—grade 3 in all of the mice joints. In the MMD+nifedipine per os (Figure 7e) and MMD+nifedipine i.a. (Figure 7f) groups, the labeling was moderate—grade 2, suggesting downregulation by nifedipine. In the healthy group, the pericellular matrix is labeled less intensively; some ECM staining occurs in the superficial layer of the cartilage–grade 1. In the nifedipine per os (Figure 7b) and nifedipine i.a. (Figure 7c) groups, the pericellular ECM of the chondrocytes is labeled less intensively than in the healthy cartilage and no staining of COLVI in the ECM was detected. These results suggest that nifedipine showed a protective effect on the damaged cartilage. The OA treatment with nifedipine per os or i.a. (MMD+nifedipine per os and MMD+nifedipine i.a.) diminished the expression of COLVI.

**Figure 8:**
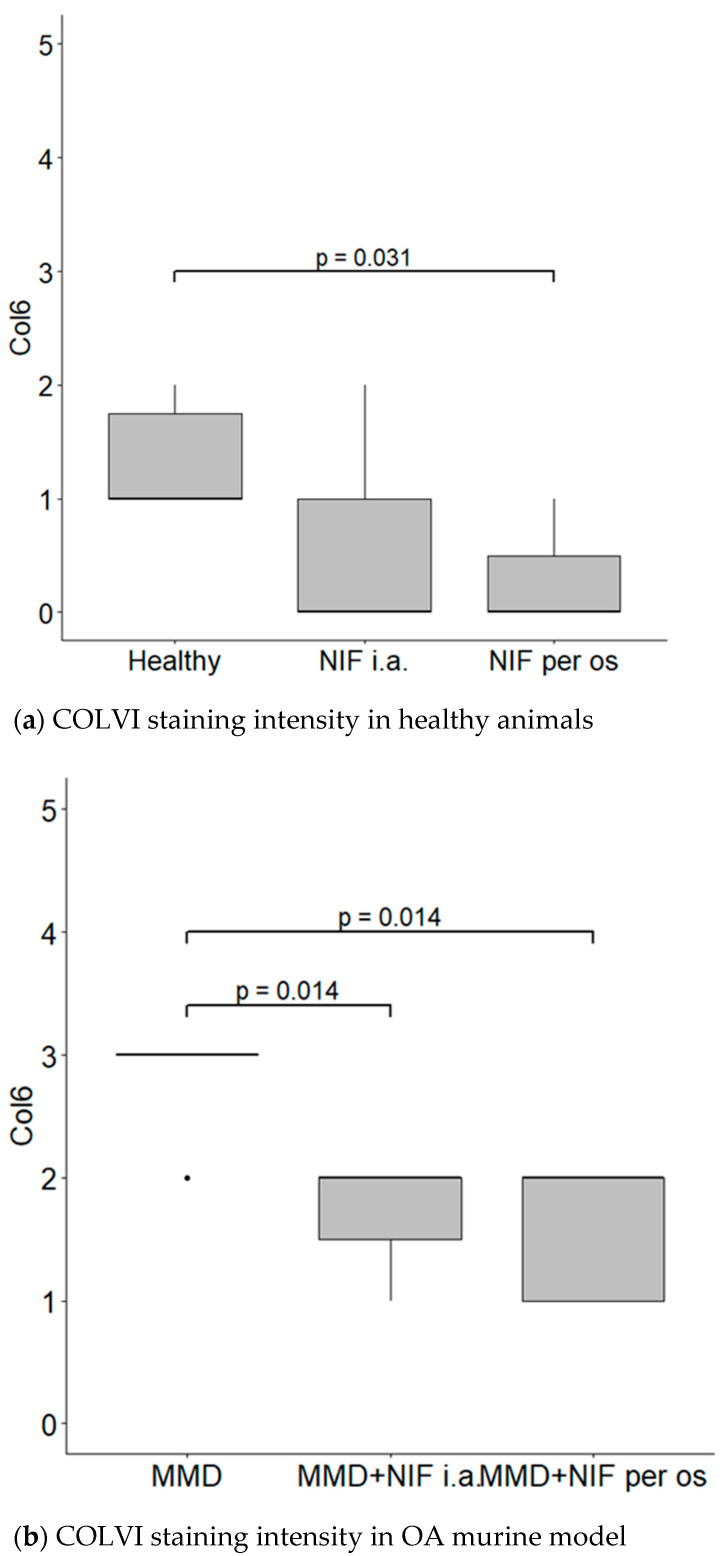
COLVI staining intensity. (**a**) COLVI staining intensity of cartilage in healthy animals. Healthy group received no treatment and had no signs of OA as evaluated using the OA staging system (ND, not detectable). NIF groups were treated with the drug for 8 weeks either per os (20 mg/kg, 5 times per week, excluding weekends), or intraarticular injection (20 mg/kg, 1 time per week). (**b**) COLVI staining intensity in OA murine model. Medial meniscus destabilization (MMD) was induced by the cutting of anterior meniscotibial and medial collateral ligaments. MMD + NIF groups were treated with NIF for 8 weeks either per os (20 mg/kg, 5 times per week, excluding weekends), or intraarticular injection (20 mg/kg, 1 time per week). Data is visualized using a boxplot, statistically significant differences are noted above with corresponding *p*-values, and plot points separated from the bars represent individual data. Wilcoxon rank sum test was used, data is considered significant at a *p* value < 0.05.

COLVI was located in the PCM of the chondrocytes in all of the groups; however, the most intensive staining was observed in the MMD group without treatment, and reached grade 3. In the healthy group, the staining of PCM was less intensive than in the MMD group. However, the COLVI staining of PCM in the groups treated with nifedipine was significantly less intensive in both the healthy animals and the OA murine model (Figure 8).

**Figure 9:**
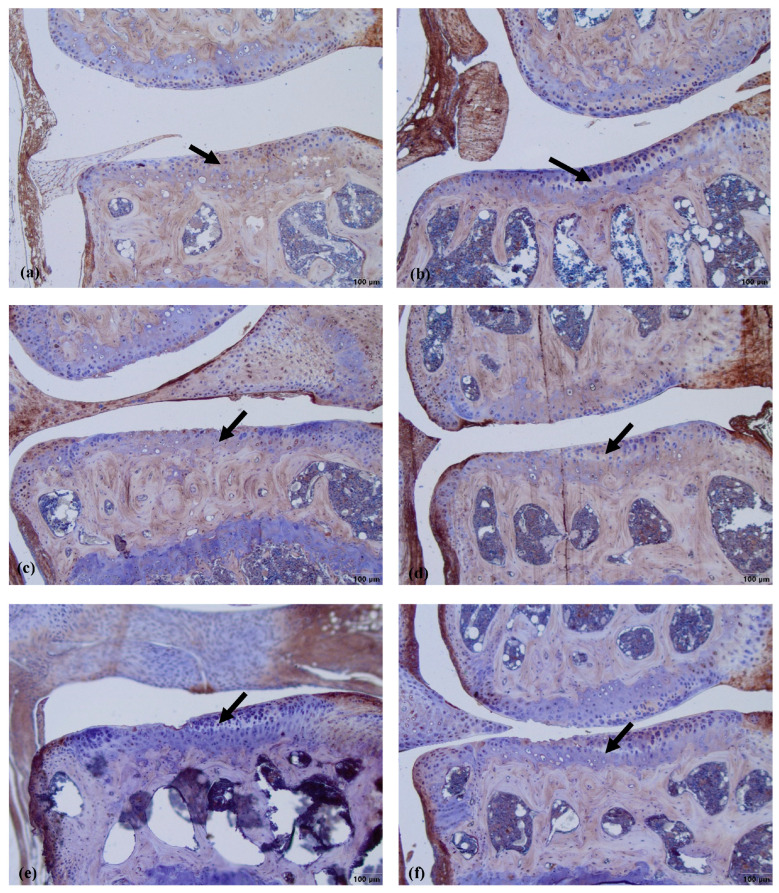
Immunostaining of CILP expression in murine cartilage. (**a**)—healthy, (**b**)—NIF per os, (**c**)—NIF i.a., (**d**)—MMD, (**e**)—MMD + NIF per os, (**f**)—MMD + NIF i.a. Arrows indicate intermediate layer of the cartilage. Magnification 10×, scale bar 100 µm.

Antibodies against CILP labeled the ECM in the middle layer of the healthy cartilage (Figure 9a). In the nifedipine per os group (Figure 9b), the staining of the intermediate layer of cartilage was reduced in comparison with the healthy group, although the cartilage does not show any dramatic structural alterations. In the nifedipine per os group (Figure 9c), the staining is dramatically reduced due to damage of the cartilage: the chondrocytes are disorganized, and it is impossible to separate distinct layers. Only light staining is visible in the upper third of the damaged cartilage. However, in the MMD group (Figure 9d), the staining of CILP remains despite the fact that the cartilage is destroyed. No staining was detected in the MMD+nifedipine per os group (Figure 9e) and MMD+nifedipine i.a. group.

We used a semi-quantitative evaluation system for CILPI staining of the intensity of the cartilage, where grade 0 stands for absent staining and grade 2 stands for intensive staining of the PCM in the cartilage.

**Figure 10:**
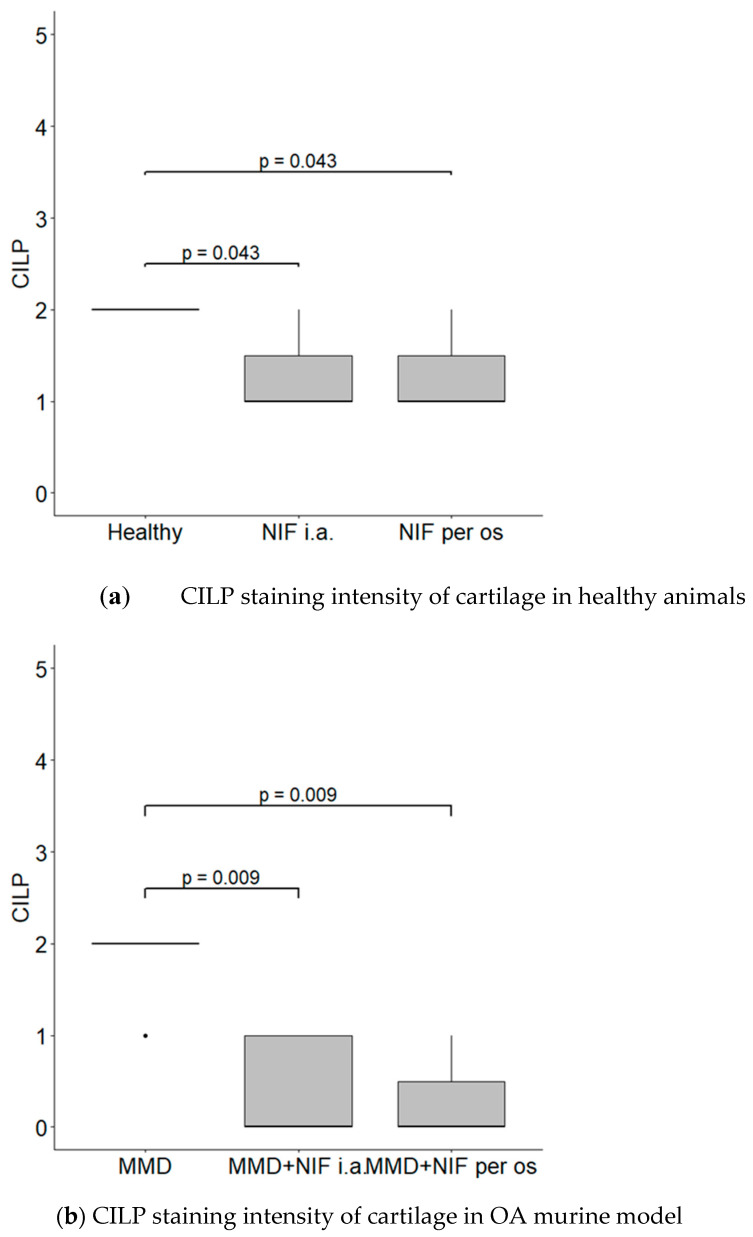
CILP staining intensity. (**a**) CILP staining intensity of cartilage in healthy animals. Healthy group received no treatment and had no signs of osteoarthritis as evaluated using the OA staging system (ND, not detectable). NIF groups were treated with the drug for 8 weeks either per os (20 mg/kg, 5 times per week, excluding weekends), or intraarticular injection (20 mg/kg, 1 time per week). (**b**) CILP staining intensity in OA murine model. Medial meniscus destabilization (MMD) was induced by the cutting of anterior meniscotibial and medial collateral ligaments. MMD + NIF groups were treated with NIF for 8 weeks either per os (20 mg/kg, 5 times per week, excluding weekends), or intraarticular injection (20 mg/kg, 1 time per week). Data is visualized using a boxplot, statistically significant differences are noted above with corresponding *p*-values, and plot points separated from the bars represent individual data. Wilcoxon rank sum test was used, data is considered significant at a *p* value < 0.05.

CILP was located in the ECM of the cartilage in all of the groups; however, the most intensive staining was observed in the healthy and MMD groups without treatment, and reached grade 2 in both of these cases. However, the CILP staining of ECM in the groups treated with nifedipine was significantly less intensive in both the healthy animals and the OA murine model (Figure 10).

**Figure 11:**
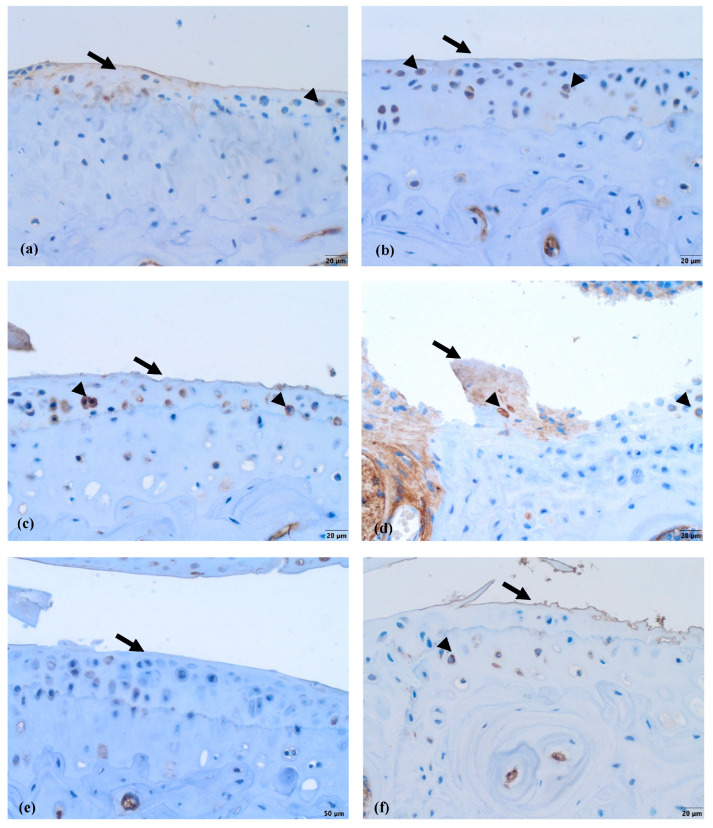
Lubricin immunohistochemical staining in murine cartilage. (**a**)—healthy, (**b**)—NIF per os, (**c**)—NIF i.a., (**d**)—MMD, (**e**)—MMD + NIF per os, (**f**)—MMD + NIF i.a. Arrows indicate surface of the cartilage, arrowheads indicate chondrocytes. Magnification 20×, scale bar 50 µm.

Lubricin is normally located in the superficial layer of the cartilage and within the cytoplasm of chondrocytes. In the control group (Figure 11a), where the cartilage was healthy, the lubricin labeling was the most intensive in comparison to the other five groups. There was reduced surface staining in the other groups; however, light cytoplasmic staining of some chondrocytes was visible in all four of the groups that received nifedipine (MMD+nifedipine per os, MMD+nifedipine i.a., nifedipine per os and nifedipine i.a.—Figure 11b,c,e,f), independently of the presence or absence of MMD. Light cytoplasmic staining of some chondrocytes and faint surface staining of the damaged area were observed in the MMD group (Figure 11d).

### 3.3. Systemic Response of the Biomarkers in Healthy and OA Mice Models after Treatment

**Figure 12:**
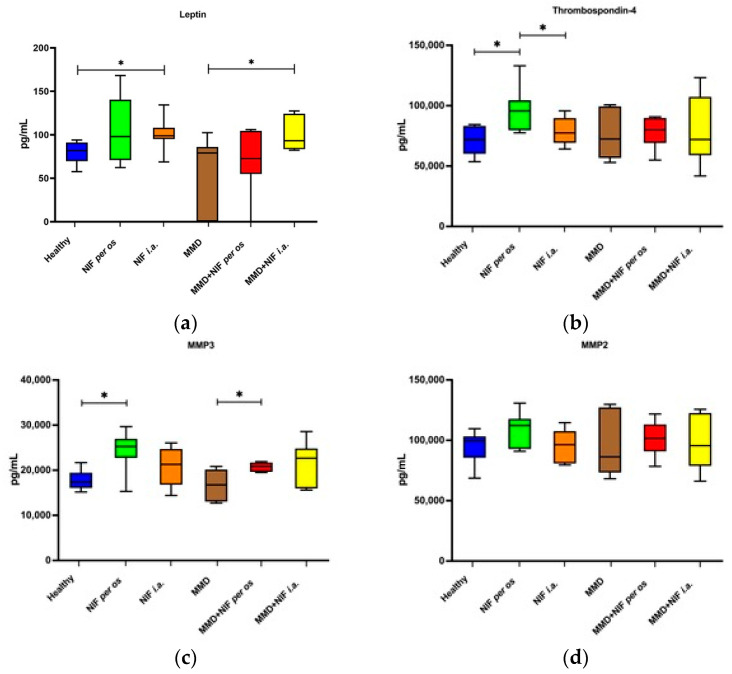
Concentration of serum biomarkers after OA model induction and treatment with NIF. (**a**)—leptin, (**b**)—thrombospondin-4, (**c**)—MMP3, (**d**)—MMP2. Data assessed by Magnetic Luminex assay. Data are presented as the interquartile range with a median value. * *p* value < 0.05.

To investigate the systemic biomarker responses, we measured the quantities of a panel of cartilage biomarkers (leptin, MMP-2, MMP-3, and TSP-4) in the serum samples from all of the treatment groups using the commercial Luminex kit, as indicated in Section 2.4. The secretion of leptin, MMP-2, MMP-3, and TSP-4 was detected in all of the groups of mice; however, nifedipine had different effects on the secretion of these factors depending on the treatment type (Figure 12a–c). The concentration of leptin in the serum was significantly higher in both of the groups that received nifedipine i.a., including the healthy mice that received nifedipine i.a. compared to the healthy control group where no treatment was administered, and the MMD group that received nifedipine i.a. compared to the untreated MMD model group (Figure 12a). The TSP-4 concentration was significantly higher only in the serum of the mice that received nifedipine per os compared to the healthy control and nifedipine i.a. groups (Figure 12b).

The serum levels of MMP-3 were increased in all of the groups that received nifedipine treatment, particularly per os, whereas they were not affected by the presence of the MMD model itself. In the MMD+nifedipine per os group, the levels of MMP-3 were significantly upregulated compared to both the healthy control group and the MMD group (Figure 12c). No differences in the MMP-2 secretion levels were detected between the groups (Figure 12d).

## 4. Discussion

OA and arterial hypertension are age-related conditions that occur side by side, and the drugs used to treat one of these diseases may have an effect on the other disease. This study was carried out to explore the potential effects of a drug used to treat arterial hypertension, VGCC inhibitor nifedipine, on the articular cartilage. MMD is a mode of meniscal incision damage that induces joint instability and subsequent OA-like changes, which, in our study, reflect severe disease stages in the OARSI semi-quantitative scoring system. The calcium L-type channel blocker nifedipineis was anticipated to exhibit multifaceted effects in OA, encompassing the regulation of inflammation, pain, and the turnover of cartilage ECM; however, the data remain controversial [23]. The action of L-Type VGCC might be associated with their activity in neuropathic pain, mediated through the spinal cord, or activities in different brain locations, although many controversies exist, mostly dependent on the model and organs involved, as well as on the specific L-type VGCC inhibitor used. Therefore, the potential effects of nifedipine on pain imply that the most likely relation is not to the mechanical loading or the local events in the joints, but rather to its systemic action, particularly in the oral administration groups. In the present study, we sought to evaluate the effects of oral and intraarticular nifedipine treatment on both healthy cartilage and surgically induced MMD, which serves as an OA model in mice [15,32]. This study has broadened our understanding of the effects of the VGCC inhibitors used for hypertension treatment on the development and progression of OA, providing new possibilities for cartilage protection. The histochemical data obtained in this study suggest that VCGG inhibitors can slightly diminish and slow down the degeneration of the OA-damaged joint: nifedipine per os induced cartilage damage, but this damage less obvious in comparison with i.a. Thus, nifedipine is likely to have adverse effects on healthy cartilage, especially in the local treatment modality. This might be associated with severe VGCC blocking and the disturbance of calcium signaling in chondrocytes under mechanical load, while normalizing calcium signaling in the overload. In this experiment, mice were treated by 20 mg/kg doses per os, according to the previously published data [52,53]. For instance, for the investigation of intestinal epithelium-specific cytochrome P450 reductase in the knockout murine model, the nifedipine dose was 10–20 mg/kg [52]; in C57BL/6 mouse virus-induced thrombocytopenia syndrome, the nifedipine dose ranged between 15 mg/kg/d and 100 mg/kg/d [53].

The immunohistochemical data further characterized the patterns of the treatment effects. Ki67 is a nuclear marker of cell proliferation, which is widely used in cancer disease diagnostics and follow-up [41]. Ki67 stains the nucleus of mitotically active chondrocytes and cryopreservation affects the proliferative and chondrogenic potential of chondrocytes [55]; however, data on how VCGG inhibitors affect chondrocytes in the settings of healthy and damaged cartilage are lacking. In our study, the healthy group chondrocytes are in the quiescent state and do not show any proliferative activity. Ki67 staining revealed differences between the cartilage in the healthy group and the other five groups, where it was similarly upregulated, suggesting that both MMD-induced damage and nifedipine treatment stimulated reparation processes in the cartilage. Also, we revealed that the healthy animal cartilage Ki67 staining was statistically different in the nifedipine per os versus nifedipine i.a. groups. This may be explained by the positive nifedipine systemic and local effect on the initially damaged cartilage (as in both groups MMD procedure was performed). Taking everything into consideration, nifedipine increases the proliferative activity of chondrocytes if used locally (i.a.) or systemically (per os); however, i.a. injections of nifedipine destroy healthy cartilage. Lubricin is located in the superficial zone of the articular cartilage and prevents damage of it due to friction [37,38]. In our study, normal lubricin expression was only found in the cartilage of the healthy group without treatment. Nifedipine treatment reduced lubricin marker labeling in some chondrocytes with light cytoplasmic staining. Nifedipine induces a reaction, with Ki67 increasing and lubricin decreasing the labeling properties in the OA model and healthy mice groups. Therefore, the chondrocyte proliferation potential and their lubrication properties are influenced by nifedipine treatment. The levels of CILP protein increase with age, as well as in the early stages of OA [34]. The expression of CILP in the healthy cartilage and in the OA cartilage showed distribution in the middle layer of the ECM. However, there was no labeling in the other four groups, where the mice were treated with nifedipine per os or i.a. This may be because nifedipine affects CILP synthesis in the cartilage. According to the ultrastructural analysis, there was evidence indicating that CILP might have an interaction with COLVI microfibrils. This suggests that CILP could play a role in facilitating connections between various components of the pericellular ECM and other parts of the ECM; furthermore, this protein showed decreased expression in a surgically induced model of OA [56]. Most relevant for the MMD model and this nifedipine treatment effect was COLVI, the staining of the pericellular matrix ECM of the damaged cartilage around the chondrocytes became more intensive and decreased accordingly in the nifedipine per os and i.a. MMD treatment groups. COLVI contributes to the mechanical properties of the pericellular ECM, chondrocyte swelling, and mechanotransduction [14]. These properties also regulate chondroprotection and regeneration in articular cartilage.

All of the biomarkers (leptin, MMP2, MMP3, TSP-4) levels were in the range of limit detection in all of the groups of this study. The concentration of leptin in the serum was significantly higher when nifedipine was administered locally in the OA model and healthy animals. Our hypothesis is that a high dose of nifedipine i.a. may influence destruction and inflammation, especially in healthy joints. This pleiotropic adipokine has different effects on the cell types in inflammation [57]. Leptin marker elevation could be related to protective anti-inflammatory effects in models of acute inflammation and during the activation of innate immune responses [42]. There was an increase in TSP-4 levels in the healthy mice nifedipine treatment groups. Therefore, the serum biomarkers were higher in the healthy groups treated with nifedipine, while only MMP-3 was significantly higher in the OA group treated with nifedipine per os compared to the untreated group. The controversial MMP-3 data are related to the nifedipine effects, but not OA damage, in the joints. Although the serum levels of MMP-2 did not reveal any changes after the administration of nifedipine, we observed an increase in MMP-3—which, to the best of our knowledge, has not previously been reported—suggesting the activation of the cartilage remodeling process. Nifedipine per os and i.a. resulted in increased levels of MMP-3, leptin, and thrombospondin-4 in mice with or without MMD, suggesting the stimulation of remodeling in the cartilage.

## 5. Conclusions

The antihypertensive drug L-type calcium channel blocker nifedipine differentially altered the cartilage remodeling and systemic responses in healthy animals and animals with MMD. Cartilage damage was identified in a medial meniscus destabilization-induced murine model of OA, as determined by the OARSI scores. Nifedipine per os and i.a. had a positive effect on the cartilage in the murine OA models. However, serial i.a. injections of nifedipine induced the deterioration of cartilage in healthy mice in vivo, suggesting its involvement in different processes in the joint. The nuclear staining of Ki67 in chondrocytes shows the proliferative activity of cells, which was higher in the MMD groups, as well as in the groups where nifedipine was used for treatment. The serum biomarkers were higher in the healthy groups treated with nifedipine, while only MMP-3 was significantly higher in the OA group treated with nifedipine per os compared to the untreated group. These preclinical data suggest that the long-term treatment of hypertension with L-type calcium channel blockers may have negative effects on cartilage tissue integrity and modulate the course of OA. In conclusion, this study demonstrates that nifedipine administration has differential effects on cartilage remodeling depending on the mode of administration and cartilage condition. Although this anti-hypertensive drug may attenuate some features of OA progression, it may also have harmful effects on healthy cartilage.

## 6. Study Limitation

A sham operated group was not included in the study design due to its compliance with the “Refinement, Reduction, Replacement” (RRR) principles, whereas in the present study design, we cannot fully exclude the possibility that some effects from the surgery itself could have occurred. On the other hand, this study is long lasting, and after 14 weeks post-surgery, when the histology examination and blood sampling was performed, residual effects of surgery itself seemed unlikely; furthermore, they were not observed in previous studies [32,58].

We did not measure the activity of inflammation and pain in the joint as the study was focused on the cartilage integrity; however, it would be interesting to investigate the effects of VGCC on the inflammatory responses and pain relief in the future studies. The action of L-Type VGCC inhibitors might be associated with their systemic activity in neuropathic pain, mediated through the spinal cord, or activities in different brain locations; therefore, a mechanical allodynia behavior test can provide additional information in future studies [59,60].

## Data Availability

Not applicable.

## Abbreviations

CILP	cartilage intermediate layer protein.
COLVI	collagen type VI.
DMSO	dimethyl sulfoxide.
ECM	extracellular matrix.
i.a.	intraarticular.
i.e.,	id est—that is.
LFC	lateral femoral condyle.
LTP	lateral tibial plateau.
MFC	medial femoral condyle.
MMD	medial meniscus destabilization.
MMP	matrix metalloproteinase.
MTP	medial tibial plateau.
NIF	nifedipine (this abbreviation was used only in figures).
OA	osteoarthritis.
OARSI	OsteoArthritis Research Society International.
TSP-4	thrombospondin-4.
VGCC	voltage-gated calcium channel.
